# An economic analysis of the impact of education on health behaviours and health outcomes in South Africa: a case of Amathole District Municipality and Buffalo City Metropolitan Municipality

**DOI:** 10.1186/s13561-025-00713-9

**Published:** 2026-01-06

**Authors:** Besuthu Hlafa, Asrat Tsegaye, Matt Dickson, Dumisani Macdonald Hompashe

**Affiliations:** 1 Economics, University of Fort Hare, 1 King Williams Town Rd, Eastern Cape, Alice, 5700 South Africa; 2 Institute for Policy Research, Bath University, Claverton, Bath, Somerset, BA2 7AY England

**Keywords:** Health behaviours1, Health outcomes2, Education3, Economic growth4

## Abstract

**Background:**

The current study investigates the economic impact of education on health behaviour and health outcomes, specifically targeting the Raymond Mhlaba and Buffalo City municipalities in the Eastern Cape region, South Africa.

**Methods:**

The study employs and modifies the National Income Dynamic Study (NIDS) data by combining all five waves to create a panel data. The study investigates an analytical objective which explored the relationship between education, health behaviours and health outcomes. This objective is addressed using a multivariate regression analysis (*multinomial logistic regression*) which offers the advantage of the ability to handle categorical outcomes with more than two categories. The current study uses marginal effects (*dy/dx*) to analyse how education affects health outcomes in each category of health outcomes.

**Results:**

The results for the probability of predictor variables belonging to category 3 “good” [coded 3 on the dummy variable (DV)] are positive and significant [dy/dx = 0.328; *p* = 0.029], showing that the marginal effects of having an undergraduate degree and belonging to category 3 are higher (33pp) than in any other category. The empirical findings suggest that education can be an important determinant of behavioral transformation and later changes in health-related outcomes.

**Conclusion:**

Thus, it is the policy implications and recommendations of this study that improving health outcomes requires more than health sector solutions. As such, given that education shapes health behaviours and health outcomes across the course of life, policies must move beyond silos and intergrade health education in South Africa’s education system in order to rule out health illiteracy as a root cause of health inequality and poor health outcomes.

## Introduction

### Background

There is a huge positive association between education and health [1]. Furthermore, the large positive correlation between health and education could be causal, or it could be that people who are more educated are future oriented and thus, invest more in health. Also, it could be that other factors that cannot be observed drive the education-health relationship and a large part of the literature aims to measure the extent of the effect of education on health and the mechanisms through which education impacts health. Education has been highlighted by scholars [2–4] to be an important mechanism for improving health and individual well-being. Feinstein et al. [5] elucidated that education reduces the need for healthcare, associated cost of dependence and human suffering because education in health is associated with improving health literacy. Furthermore, the impact that education has on health behaviours and outcomes is important such that education helps promotion and sustainability of positive life choices and healthy lifestyles, supporting and nurturing human development, relationships, personal development, family relations and community well-being. There are different definitions of education from different scholars, coming from different schools of thought. *Aristotle* defines education as “the process of training man to fulfil his aim by exercising all the faculties to the fullest extent as a member of society”; while Gregory (2002) posited that education is concerned with equipping the mind to make sense of the physical, social and the cultural world. In this study, education is defined broadly as the personal, cognitive, and social skills that govern the ability to access, understand and use information to promote and sustain good health as posited by Nutbeam (2006), which is also closely related to the definition by Gregory (2002). Education is thus a measure of the individual’s ability to read, comprehend and act on medical instructions.

Health behaviours can be clearly defined as individual actions that could influence individual health status. These individual actions can either lead to improved health status such as maintain a healthy diet and physical activity, or they could lead to deteriorating health which increases the burden of disease such as smoking, alcohol abuse, and among many, risky sexual activities. Barnes et al. [6] posited that health behavioural change has been promoted as a potential health intervention strategy that could help change deteriorating health outcomes for the better. Brunello et al. [3] defined health outcome as a *change* resulting from antecedent healthcare. It is therefore important to take note of the word ‘*change*’ in the definition, which can either imply improvement or deterioration of the related outcome. The responsiveness of an outcome measure is fully dependent on its sensitivity to behavioural, clinically meaningful change. Thus, the changes in outcomes are fully dependent on the changes in health-related behaviour. Therefore, the responsiveness of an outcome measurement cannot be evaluated separately from its reliability.

The measurement of these outcomes can be clinical (physical examination, laboratory testing), self-reported or observed (movements of fluctuations seen by a health professional). Health outcomes that have been proven to be, to some extent, affected by health-related behaviour are *life expectancy* and *mortality*. Life expectancy at birth is an estimate of how long a person born today will live on average if the current mortality rate in each age group does not change throughout life [7]. The justification behind using life expectancy as an outcome measure is based on the assumption that health and literacy have a positive contribution towards longevity [8]. To further explain this, life expectancy is affected by many factors including: Socio-economic status which comprises employment, education, income and economic well-being; the quality of healthcare system and the quality of access to it; health behaviours such as smoking and heavy drinking, poor eating habits and lack of exercise; social factors; genetic factors; and environmental factors [9]. These factors also include congested homes, lack of safe drinking water, and proper hygiene. More so, education thus informs behavioural change. Health behaviour is a determinant of longevity through which if it is positive, lifespan is increased and thus increasing related health outcomes (*life expectancy*).

Mortality rates are a useful measure of the overall health status of a population to measure the improvement over time. According to [9], the COVID-19 pandemic resulted in a significant increase in crude death rate (CDR) from 8.7 deaths per 1000 people in 2020 to 11.6 deaths per 1000 people in 2021. This significant increase in death rates meant a drop in the 2021 estimates of life expectancy at birth for South Africans. Mortality is linked to life expectancy as an increase in life expectancy suggests a decline in mortality rates. For the reported period of time (2020–2021), COVID-19 has not been the only cause of increased mortality rates, as other deaths resulted from unimproved health behaviours such as alcohol abuse, which led to numerous fatalities [2].

### The relationship between education, health behaviours and health outcomes

**Fig. 1:**
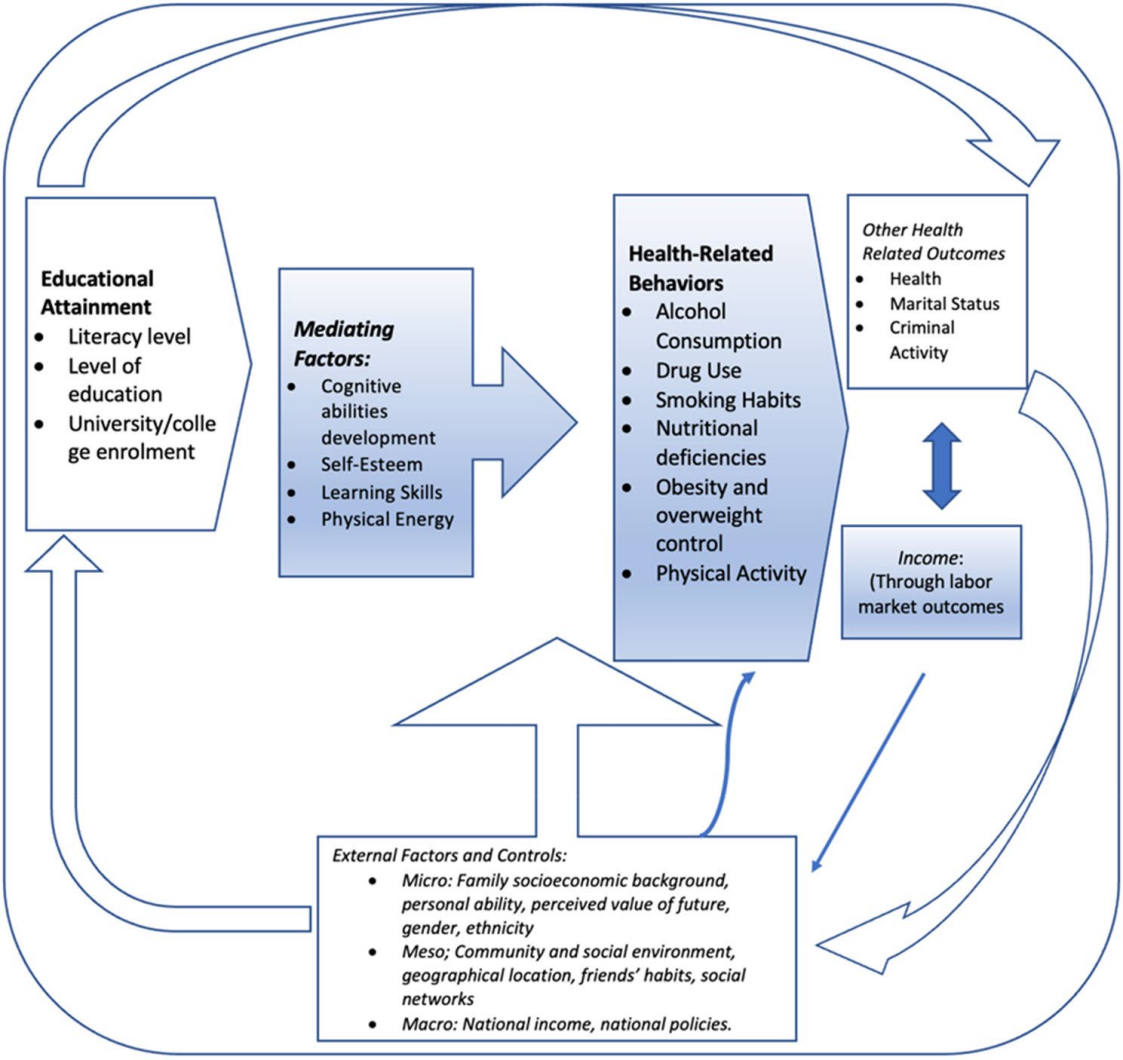
Analytical work of the relationship between education and health

The relationship between education, health behaviours and health outcomes is easy to understand. However, this relationship has been subject to a lot of debate and extensive research given that the direction of I influence, mechanisms and implications have contrasting views. Some scholars [10] allude that the relationship comes from health to education while others [11] argue that the demand for health comes from a position of knowledge and literacy, thus they assert that the relationship comes from education to health and continues in a circular mode. Also, it is quite complex to understand how education, health behaviours and health outcomes affect each other given that there are other unobserved factors such as income which can be major determinants for these variables. The Michael Grossman Model (MGM) of health production (1972) simplifies this relationship, showing how the dependence of these variables to one another can lead to a better economic output. There are three documented mechanisms that potentially drive the relationship between education and health: Education may determine health; one or more factors may simultaneously determine both education and health (socioeconomic status); health may determine education. This study follows the first mechanism and to support this, an inverse analytical framework ofSuhrcke and de Paz Nieves [10] is presented in Fig. 1 below:

The analytical framework presented in Fig. 1 above was adopted and modified from the study of Suhrcke and de Paz Nieves [10]. According to the latter, the predominant view appears to be that the first mechanism of the relationship between education and health is primarily driving the association, particularly in high-income countries. This predominance is also based on the measurement of education in these high-income countries. The current study presents an entirely different approach to the definition of education, which might enhance the first mechanism and potentially make it the only mechanism for all economies. The link between education and health, where education is primarily the driver of the relationship between the two, has different potential explanations, most of which suggest that though other mechanisms may exist, the first mechanism provides a stronger motivation than the rest. The impact of education on prospects, through changed behaviors and intergenerational transmission of inequalities begins with proper educational attainment. Education attainment for children, along with health and external factors account for differences in employment status and financial stability when they become adults, as well as their health outcomes and overall future prospects in a self-reinforcing cycle [10].

### Literature review

The human capital and the health capital theories reviewed in this study addressed the relatedness of education and health and also introduced longevity, which is the time length over which the health and skill capital investment benefits can be reaped. From the reviewed theories, we concluded that investing in education (skill capital) alone is not ideal because one’s life may be cut short before they can truly enjoy the investment benefits of education because of deteriorating health. Also, investing is health alone is not enough if one cannot financially support the extended life due to lack of skill capital. Thus, the three theories combined posit that investment in both skill capital and health-capital in due time favors economic growth and also increases the duration of the time in which the investment benefit on health and education can be enjoyed. Theoretically, particularly in South Africa and the rest of the African countries, while there are studies which investigate the association between education and health outcomes, there are no studies which investigate district-level economic analysis while also suggesting the direction of influence as indicated in Fig. 1. Also, there are no similar studies in the chosen geographical area where the study was conducted, in Eastern Cape. The identified gap thus justifies the original research contribution, both to academic knowledge and policy formulation. Also, given that there are not studies on the subject matter in South Africa, particularly in the Eastern Cape, the triangulation method which this study employed makes it a unique and a significant study as this the only study which employs the education variable to examine literacy levels of the participants, suggesting that higher literacy levels should inform good healthy behaviour and thus good health outcomes.

While many studies (Viinikainen et al. 2022; Ohonba et al. 2019; Davies et al. [12] measure education by *Years of schooling*, and while this is important for marginal effects comparability and straightforward interpretation, these studies assume linearity and ignore the quality of education. The current study conceptualizes education to measure literacy levels (*measures the ability of an individual to read*,* comprehend and act on medical/doctors’ instructions*), which also helps analyse marginal effects and can be comparable. It also does not assume linearity and does not ignore quality of education.

Much has been done on the influence of education on health and the impact of education on behavior separately. Very few studies [13] have come close to investigating the impact that education has on health behavior to improve health outcomes. Some studies have clearly shown that there are health outcomes that cannot be controlled by the level of educational attainment, nor literacy. Thus, for health outcomes such as mortality rate, education proves to be an insignificant impact, this is based on the fact that most deaths are not resulting from poor education attainment background. This research thus focuses on the health outcomes that can be controlled or influenced by education such as smoking and alcohol consumption.

Studies which investigated the causal effects of education on health behaviour and health outcome [3, 14]; Davies, Dickson, Smith, et al., [12]) from both developed and developing worlds presented contradicting results. The observation of this study, based on the difference in the results of the above-mentioned studies was that geographical setting and status of a nation played an important role in the outcomes determined by these various studies. Those that were conducted in a developed setting proved that an additional year of schooling positively affected health outcomes, while those in the developing countries proved no correlation between the two variables. In most of the LMICs, there is no educational program (primary or tertiary) that specifically targets the awareness and improvement of health outcomes which will result from healthy choices. Most unhealthy choices and decisions in the poorest communities were resulting from childhood adverse behaviours which had a multiplier effect on the children who grew up in such circumstances.

From the reviewed literature, both developed and developing, it is clear that preventive intervention educational programs are necessary impact on bad healthy behaviours. These interventions should thus be clear and precise to the targeted behaviours to fully have an impact.

## Data and methods

The data source was from the *National Income Dynamics Study (NIDS)*, South Africa’s first panel study that sought to track and understand the gnawing effects of South Africa’s stubborn poverty [15] by following the lives of the same 28 000 South African households since 2008. The data consists of five waves which were collected between 2008 and 2017. NIDS (2024) asserted that approximately every two years, the same households of 28 000 South Africans were re-interviewed by highly trained field workers to find out what - *if anything* - had changed for them since their last interview. In every wave, there are a series of questionnaires – *Adult*,* Child and Household* – that are used as data collection instruments from these households.

To address the main objective of the study, the datasets from all five waves were merged and appended into one longitudinal dataset, sorted by a unique identifier referred to as a personal identifier (*pid*) across all waves. From the available data, some variables were grouped into categories and some recoded for ease of analysis and discussion. Given that the geographical area of interest for investigating the impact of education on health behaviour and health outcomes as mentioned in this study is *Buffalo City Metropolitan Municipality (BCM)* and *Amathole District Municipality (DC12)*, the NIDS data was further reduced, omitting all other municipalities from investigation but the ones who are of interest to the study. As a result, the total number of observations measured by *household identifier (hhid)* and by *pid* is 713. Of the 713, 288 were from BCM and 425 from DC12.

For this research, we utilized secondary data from South Africa’s first national panel study, the National Income Dynamics Study (NIDS), to investigate the relationship between education and health behaviours and outcomes. To account for other important individual characteristics that might confound this relationship—such as age, gender, ethnicity, and family background—we employed multivariate regression techniques.

Given that several of the outcome variables in this study (for example, self-rated health, smoking, and alcohol consumption) are categorical or binary, we estimated limited dependent variable models, specifically logit specifications, rather than standard linear regressions. These models express the probability of observing a given health outcome as a function of education and other covariates. The basic model can be represented as:

1 $$\:P\left({y}_{i}=1\right)=F(\alpha\:+\:\beta\:{Ed}_{i}+\:{\theta\:}_{1}{Inc}_{i}\:\:{{X}^{{\prime\:}}}_{i}\gamma\:)$$

where $\:{y}_{i}$ represents the health outcome for individual $\:i$, $\:{Ed}_{i}$ represents educational attainment which measures literacy levels, $\:{X}_{i}$ is a vector of control variables, and $\:F$ is a cumulative distribution function. The error term is thus implicit in the function in the form of $\:F$.

To explore the potential mediation effects, the analysis also considered the extent to which education influences health outcomes indirectly through its effects on health behaviours such as smoking and alcohol consumption. The mediating models were. Estimated as:

2 $$\:P\left({S}_{i}=1\right)=F({\alpha\:}_{1}+\:\lambda\:{Ed}_{i}+\:{\theta\:}_{1}{Inc}_{i})$$

3 $$\:P\left({A}_{i}=1\right)=F({\alpha\:}_{2}+{\gamma\:Ed}_{i}+{\theta\:}_{2}{Inc}_{i})$$

where $\:{S}_{i}$ and $\:{A}_{i}$ are smoking and alcohol behaviours, respectively. Then, to assess whether, and to what extent does education mediate the effect, these health behaviours were incorporated into the main health outcome model. This can now be written as:

4 $$\:P\left({y}_{i}=1\right)=F({\phi\:}_{0}+{\phi\:}_{1}{S}_{i}+{\phi\:}_{2}{A}_{i}+{\pi\:Ed}_{i}+{\theta\:Inc}_{i}+{X}_{i}^{{\prime\:}}\delta\:$$

Comparing the estimated coefficients on education across these models allows for an assessment of the extent to which the influence of education on health outcomes is mediated to changing behaviours. Further decomposition analyses were conducted to explore other mechanisms through which education impacts health, while acknowledging that the results rely heavily on the assumption of selected observable characteristics. This approach thus appreciates and provides meaningful insights to into both the direct effect of education on health and the indirect pathways operating through health-related behaviours. This offers useful evidence for integrated public health and education policy design.

The application of the modelling framework to the chosen geographical area (*Eastern Cape*).

## Results and discussion

### Results for *healthy behaviour* as an outcome variable

The study estimated a multinomial logistic model for healthy behaviour (*h_b)* as an outcome variable. For a case-by-case analysis and a clear interpretation of the regression results according to the categories of healthy behaviour, the study interpreted the probability outcomes (*the probability of belonging to each category*) presented in Table 1 (*h_b* = = worst); (*h_b* = = bad); (*h_b* = = good); and (*h_b* = = best), respectively. The results for the probability of predictor variables belonging to all categories: [“worst”, coded 1 on the dummy variable (DV); “bad”, coded 2 on the dummy variable (DV); “good”, coded 3 on the dummy variable (DV) and “best” [coded 4 on the dummy variable (DV)] are presented in Table 1.

Table 1 shows the predictor variable as *edlevel*, with 5 levels of education. The average marginal effects for primary [dy/dx = 0.069; *p* = 0.283]; secondary [dy/dx = 0.078; se = 0.085; *p* = 0.355]; and post-secondary [dy/dx = 0.005; *p* = 0.964] for category 1 are positive and statistically insignificant, meaning that compared to no education, having primary, secondary or post-secondary education does not increase the chances of being in the worst healthy behavioural state. The results also indicate an inverse relationship between belonging in the worst category for someone with an undergraduate degree: compared with someone with no schooling, having an undergraduate degree substantially reduces the chance of being in the worst health category [dy/dx= −0.236; *p* = 0.000]. The average marginal effects for incomplete secondary [dy/dx= −0.035; *p* = 0.598] is negative and insignificant. The results for undergraduate indicate that a negative significant relationship exists between having an undergraduate degree and reporting a worst healthy behaviour. Thus, it is less likely (23pp) for someone with an undergraduate degree to report a worst healthy behaviour or to belonging to the “*worst*” category than someone with no schooling.

The next regression output in Table 1, columns 2 and 3 is for the marginal effects of being female and belonging to category 1. The average marginal effects for *female* [dy/dx = 0.053; *p* = 0.162] are positive and insignificant, meaning that the likelihood of belonging to category 1 for female is 5% compared to male. Again, taking a closer look at the average marginal effects of the *lhhincome* under category 1 [dy/dx= −0.127; *p* = 0.000], it is negative and significant, which means that as income increases by 100% it makes it less likely (13pp) for the individual belongs to category 1 or reports a *worst* healthy behaviour.

The results for the probability of predictor variables belonging to category 2 “bad” [coded 2 on the dummy variable (DV)] are presented in column 3 and 4 of Table 1. For education levels, the variable *undergraduate* [dy/dx= −0.076; *p* = 0.047] indicates a negative and significant association between belonging to category 2 and having an undergraduate degree. This can be interpreted to indicate that a person with an undergraduate degree is less likely (8pp) to belong to category 2. The *post-secondary* level of education is showing negative average marginal effects but is insignificant [dy/dx= −0.006; *p* = 0.942]. This means that people with a post-secondary level of education are less likely (0.6pp) to belong to category 2.

According to Boerma et al. (2016), women reported significantly poorer health than man in all ages. However, the results reported for *female* in Table 1 [dy/dx= −0.147; *p* = 0.000] indicate that females are less likely (15pp) to belong to category 2 than men and are more likely (15pp) to belong to category 4 (best) [dy/dx= −0.150; *p* = 0.000] as indicated in Table 1, columns 8 and 9. Although the results are unexpected, given women’s frequency for utilising health facilities, the results seem to be consistent with Mathentamo et al. [15], where young women generally reported more positive subjective well-being than young men. Although the variable of comparison (*healthy behaviour (h_b) and Subjective well-being (SWB)* between this study and that of Mathentamo et al. [15] are not the same, they both represent health outcomes.

Also, although statistically insignificant [dy/dx= −0.016; *p* = 0.218], a 1 unit increase in income makes it less likely (2%) for an individual with this increase to belong to category 2 with respect to healthy behaviour.

The results for the probability of predictor variables belonging to category 3 “good” [coded 3 on the dummy variable (DV)] are presented in columns 6 and 7. While most of the slopes for *edlevel* are positive, there is only one positive and significant slope [dy/dx = 0.328; *p* = 0.029] and the marginal effects of having an undergraduate degree and belonging to category 3 are higher (33pp) than in any other category. For a unit increase in education level (*undergraduate*), the likelihood of falling into category 3 is increased by 33pp and the results are significant. Put simply, it is highly likely for a person with an undergraduate degree as their highest level of education to report good healthy behaviour. These results suggest a positive relationship between education and health outcomes. A larger percentage of likelihood between the same group of individuals (*undergraduate*) for all categories (1–4) is positive and significant [dy/dx = 0.328; *p* = 0.029] in category 3 “good”.

The conclusion that can be drawn from the category 4 marginal effect results pertaining to having an undergraduate degree is that education positively influences behaviour. Whether this behaviour (for the IV undergraduate) positively affects health status as a health outcome variable is discussed with the regression results where *health_status* is analysed as a health outcome variable.

Columns 8 and 9 of Table 1 also indicate that being female is less likely (5.5pp) to be associated with reporting good healthy behaviour or falling into category 3. The slope for *female* [dy/dx= −0.055; *p* = 0.203], meaning that for a unit increase in the IV, the likelihood of falling into category 3 decreases by 6%. Again, as the results for category 4 indicate, being female is more likely to be associated with reporting the best healthy behaviour, maybe a consequence of most females in the dataset not smoking (96%), not drinking (92%), and eating fruit and vegetables (39%). While 81% of the females in the dataset do not exercise, they spend more money on medical professionals and medical supplies than men.

From the results presented in Table 1, it can be noted that many coefficients for the IV *edlevel* (education) have high p-values (> 0.1), indicating no strong statistical relationship between education and the dependent variable (*h_b*). To assess the possible causes of these results, and to rule out the possibility of multicollinearity between education and income (*hhincome*), we tested for variance inflation factors (VIFs) and the results showed a VIF of 1.20, suggesting that there is no multicollinearity between the variables. Also, the extremely large standard errors for some of the education variables like “undergraduate” and “other” suggest few observations, which can lead to lack of statistical power to detect true effect. The other possibility for these results can be ‘true lack of association’, meaning that perhaps education genuinely does not affect particular health behaviours such as smoking and physical activity. The latter also presents the possibility of homogeneity for the group being studied. Within the dataset, people with different education levels may behave similarly when it comes to the healthy behaviours in question. These results suggest that targeting education alone may not be effective in improving health behaviours within this group.

**Table 1:** Probability prediction for outcomes for categories: [h_b = = worst]; [h_b = = bad]; [h_b = = good]; [h_b = = best]

	Delta-method									
	Pr(Ih_b = worst) dy/dx	z		Pr(Ih_b = bad) dy/dx	z	Pr(Ih_b = good) dy/dx		z	Pr(Ih_b = best) dy/dx	z
edlevel										
primary	0.069	0.283		0.049	0.293	−0.072		0.346	−0.046	0.553
incomplete secondary	−0.035	0.598		−0.009	0.844	0.044		0.603	0.000	1.000
secondary	0.078	0.355		0.063	0.278	−0.111		0.226	−0.030	0.747
post-secondary	0.005	0.964		−0.006	0.942	0.024		0.846	−0.023	0.830
undergraduate	−0.236	0.000		−0.076	0.047	0.328		0.029	−0.017	0.911
other	−0.236	0.000		−0.076	0.047	0.211		0.489	0.100	0.743
female	0.053	0.162		−0.147	0.000	−0.055		0.203	0.150	0.000
race_1	1.869	0.988		0.696	0.993	−1.583		0.981	−0.982	0.985
age	0.001	0.453		−0.001	0.326	0.002		0.146	−0.002	0.106

Note: dy/dx for factor levels is the discrete change from the base level

### Results for *health status* as an outcome variable

Health status is a categorical variable with 5 categories: 1 “Poor”, 2 “Fair”, 3 “Good”, 4 “Very Good”, 5 “Excellent”. For the analysis of statistical results, the study used the categorical values and names (i.e. 1 or Poor) interchangeably. The first predictor variable displayed is *edlevel*, and for this variable only *undergraduate* [dy/dx = 0.440; *p* = 0.000] is statistically significant. The results suggest that having an undergraduate degree significantly increases the likelihood of reporting poor health (4pp), whereas higher degrees reduce this likelihood to 4.6pp. This means that for a unit increase in this variable (*undergraduate*), the likelihood of falling into category 1 increases by 44pp. Although literature on health behaviour and outcomes of university/college students is thin, these results are consistent with the findings of Von Ah, et al. (2004) and Helmer and Mikolajczky, (2012). The latter asserted that students are engaged in more unhealthy behaviours especially given that first time entrants experience greater freedom and control over their lifestyles than they ever did before, thus potentially engaging in various unhealthy and risky health behaviours (Von Ah, et al. 2004). Another statistically significant variable in Table 2 under category 1 is *age* [dy/dx= −0.007; *p* = 0.000]. This means that the likelihood of belonging into category 1 for every unit increase in the independent variable *age* are 0.7pp less. Again, this is consistent with Mathentamo et al. [15]:7), where the authors found that “as health deteriorates, it becomes a significant contributor to subjective well-being (SWB)”. The marginal effects for the rest of the education variables carried the correct sign but remain statistically insignificant. The same can be concluded about gender (female) and race (race_1).

Category 2 results, presented in columns 4 and 5, also confirm the latter results for *age*. The average marginal effects for the IV *age* [dy/dx= −0.002; *p* = 0.018] are negative and significant, meaning that for a unit increase in the IV, the likelihood of belonging to category 2 decreases by 0.2pp.

The IV *undergraduate* in Table 2 now appears to be negative and significant, contrary to what it was for category 1. The results for *undergraduate* shown for both categories 1 and 2 do not contradict each other. The likelihood of belonging to category 1 for this variable is stronger (44pp) while the likelihood of belonging to category 2 is weaker (17%). This means that the likelihood for people with an undergraduate degree to belong to category 1 is stronger, also supported by the fact that the slope for *undergraduate* in category 2 is negative and significant, meaning that the likelihood of belonging to category 2 with an undergraduate degree is 17% less. Although statistically insignificant, higher literacy variables (post-secondary & higher degree) for *edlevel* are negative, meaning that for a unit increase in the IV, the likelihood of belonging to category 2 is less by 4.9% and 2.7% respectively. These results can be interpreted to indicated that there is a weaker relationship between having these qualifications and belonging to category 2 with respect to subjective health status.

**Table 2:** Probability prediction for outcomes: 1 [health_status = = Poor]; 2 [health_status = = Fair]; 3 [health_status = = Good]; 4 [health_status = = Very Good]; 5 [health_status = = Excellent]

	Delta-method									
	Pr(health_status = Poor) dy/dx	z	Pr(health_status = Fair) dy/dx	z	Pr(health_status = Good) dy/dx	z	Pr(health_status = Very Good) dy/dx	z	Pr(health_status = Excellent) dy/dx	z
edlevel primary	−0.063	−0.200	0.053	0.394	−0.011	0.854	−0.001	0.986	0.022	0.260
incomplete secondary	0.010	−0.127	0.029	0.643	0.003	0.960	−0.020	0.560	−0.022	0.246
secondary	0.088	−0.063	0.061	0.392	−0.014	0.842	−0.093	0.014	−0.042	0.007
post-secondary	0.134	−0.024	−0.049	0.479	−0.079	0.288	−0.035	0.465	0.028	0.477
undergraduate	0.440	0.203	−0.179	0.013	−0.077	0.497	−0.142	0.000	−0.042	0.007
higher degree	0.046	−0.208	−0.027	0.811	−0.035	0.819	−0.142	0.000	0.158	0.309
other	0.475	0.065	−0.222	0.000	−0.333	0.000	0.123	0.540	−0.042	0.007
1.female	−0.047	−0.099	0.025	0.346	0.029	0.307	−0.001	0.969	−0.006	0.618
1.race_1	0.065	−0.046	−0.081	0.216	0.048	0.464	0.021	0.650	−0.053	0.378
age	−0.007	−0.009	−0.002	0.018	0.003	0.000	0.005	0.000	0.002	0.001
lhhincome	0.012	−0.014	0.026	0.067	−0.018	0.249	−0.009	0.430	−0.011	0.115

Note: dy/dx for factor levels is the discrete change from the base level

For the IV *female*, there is likelihood that being female is associated with belonging to category 2 (fair) and 3 (*good*) as indicated in column 4 & 5 and again in column 6 & 7, with 2.5pp and 2.9pp likelihood respectively. Again, the relationship in both cases is statistically insignificant.

Category 3 (*good*), presented in column 6 & 7 has only one significant variable, *age* [dy/dx = 0.003; *p* = 0.000] with a *p-value* of 0.000 and positive average marginal effects of 0.3. For a unit increase in age, the log-odds of belonging in category 3 increase by 0.3%. This is consistent with the findings in Mathentamo et al. [15] where increase in age was found to be associated with increased SWB, which in the case of this study can translate to increase in age is associated with reporting good health or belonging to category 3.

The probabilities of the predictor variables belonging to category 4 are presented in column 8 & 9. There are four variables with statistically significant results presented in this section. The first variable under *edlevel* is *secondary* [dy/dx= −0.093; *p* = 0.014], followed by *undergraduate* [dy/dx= −0.142; *p* = 0.000] and *higher degree* [dy/dx= −0.142; *p* = 0.000]. For a 1 unit increase in the categories of *eldlevel*, the likelihood of belonging to category 4 decrease by 9.3pp, 14.2pp and another 14.2pp for *secondary*, *undergraduate*, and *higher degree*, respectively. The expectation from this study was that increase in education level/literacy had a significant impact on improving health behaviours. The results presented here suggest that there is less likelihood for people with the mentioned levels of education to report a *very good* health status. Average marginal effects for *undergraduate* and *higher degree* for category 4 are identical. To investigate why this may be, the study stratified the data by *edlevel* and summarized *health_status*. The study found that for *undergraduate* and *higher degree*, there were no responses or no one from the dataset reported to belong to category 4 from the 2 levels of education respectively. Also, out of 143 observations for secondary schooling level, only 2.8% of the population reported to have a *very good* health status. The fourth statistically significant results under category 4 are for *age*. The slope for age is positive, confirming the same results presented in the preceding section. While the expectation was that as age increases health deteriorates, the results here show that for every unit increase in *age*, the likelihood of reporting a *very good* health status increase by 0.5pp.

The results for the probability of reporting an excellent health status or belonging to category 5 are presented in columns 10 & 11. Once again, the average marginal effects for secondary and undergraduate are identical [dy/dx= −0.042; *p* = 0.007], a signal that there were no participants with these levels of education who reported an excellent health status. For a unit increase in *age*, the likelihood of belonging to category 5 increase by 0.2pp. Again, the results for *age* have been consistently reporting that the older people become, the more they are likely to report good health status which is also reported by [15].

Most of the results presented in this section for the marginal effects, the independent variables carried the expected signs for their slopes, signaling that education does have an impact of both health behavior and health outcomes, however, the relationship is weak given its statistical insignificance.

## Conclusion and recommendations

This study advocates for joint investment in education and health for the purpose of the economic benefit that comes with such investment. It has also been revealed in this study that returns on education investment have been gradually declining, given the lack of employment even for those that have obtained highest level of education. For the proposed joint investment in education and health to have impact, certain structural changes in government need to be implemented. Thus, it is the recommendation of this study that our education system be transformed such that quality is prioritized more than attainment. Years of schooling for a developing country like South Africa alone cannot fully inform education’s impact on outcomes. Already, Spaull (2015) posited that low quality education resulting from overcrowded classrooms; undertrained teachers; poor infrastructure and language barriers in South Africa can easily lead to a poverty trap, given the possibility of lacking skills required by employers which then leads to failed education investment and unemployment. To prioritize quality helps reduce the burden of the latter. Secondly, without health literacy in the education system, education in its current form cannot be expected to have an impact in improving health or the impact of education on health cannot be fully appreciated. It is the recommendation of this study that health literacy be integrated into the South African education by encouraging collaboration between education and health departments to created integrated programs. The initiatives such as NIDS can be more effective to study the patterns of behaviour for individuals if collaborative longitudinal studies can be implemented. This would allow the links between education and health outcomes to unfold and be carefully studied in order to initiate transformation.

Returns on education investment are financial, while returns on health investment are longevity to enjoy the benefits of investment in education. Unemployment among youth was estimated at 45.5% by Stats SA (2024), which was considerably higher than the national unemployment rate of 31.9%. The qualitative results also revealed that some of the youth engaged into smoking and drinking behaviours as a result of the unemployment burden. It is the recommendation of this study that researchers need to investigate how quality of higher education gives rise to such high unemployment rates. Also, the indoctrination of government dependency for jobs among youth should be carefully studied and resolved. Lastly, the education and health policies often operate in silos, which would defeat the purpose of having a joint education-health investment. This study recommends investigating the impact of inter-department (*health and education*) policies for the education-health investment to impact positive returns on investment and possible economic benefit. Endogeneity.

## Data Availability

The data used in this study can be accessed from DataFirst – *National Income Dynamic Study* – under University of Cape Town’s (UCT) website.
